# Natural Language Processing of Clinical Notes for Cancer Research and Patient Care Prior to Widespread Adoption of Generative AI: Scoping Review

**DOI:** 10.2196/73481

**Published:** 2026-05-14

**Authors:** Alfred B Kayira, Hadeel R A Elyazori, Kevin Lybarger, Fiona M Walter, Claude Chelala, Garth Funston

**Affiliations:** 1 Centre for Cancer Screening, Prevention, and Early Diagnosis, Wolfson Institute of Population Health, Queen Mary University of London, Charterhouse Square, London, EC1M 6BQ, United Kingdom, 44 7415302686; 2 Department of Information Sciences and Technology, College of Engineering & Computing, George Mason University, Fairfax, VA, United States; 3 Barts Cancer Institute, Queen Mary University of London, London, United Kingdom

**Keywords:** natural language processing, clinical notes, electronic health records, clinical NLP challenges, cancer, scoping review

## Abstract

**Background:**

Clinical notes are the most abundant data type within electronic health records; however, their highly unstructured format presents significant challenges for supervised natural language processing (NLP) methods. The NLP community is increasingly adapting large language models to analyze clinical notes, achieving strong performance and generalizability with minimal task-specific fine-tuning. We conducted a scoping review of NLP methods applied to clinical notes prior to widespread adoption of generative artificial intelligence (AI) to establish a pre–large language model methodological baseline, showcase potential clinical utility, and highlight key challenges and limitations of extractive, supervised techniques that generative AI approaches may need to overcome.

**Objective:**

This review aimed (1) to characterize the clinical notes used, (2) to identify NLP techniques used to analyze these notes, (3) to determine the clinical applications of NLP in cancer research and patient care, and (4) to highlight challenges and limitations of traditional pregenerative AI methods.

**Methods:**

We systematically searched MEDLINE, Embase, Scopus, and Web of Science for English-language studies published from January 1, 2014, to March 8, 2024. Retrieved references were imported into Covidence, a web-based platform that streamlines management of reviews. Two authors (ABK and HRAE) independently screened studies for eligibility and extracted data using a predefined data extraction template.

**Results:**

A total of 226 studies were included in the review. Research using NLP to derive insights from clinical notes grew significantly, from 4 studies in 2014 to 43 in 2023. NLP methods have evolved from predominantly rule-based and ontology-driven approaches (2014-2017) to hybrid approaches that combine these with deep neural models such as Bidirectional Encoder Representations from Transformers (2018-2024). Most studies (161/226, 71.2%) developed their systems using small, single-institution datasets. Supervised learning approaches with manually annotated corpora were predominant (181/226, 80.1%). Most studies (174/226, 77%) focused on information extraction, with a few applying the extracted data to downstream tasks such as diagnostic and prognostic classification. Clinical domain pretrained models outperformed general domain pretrained models in the majority (11/16, 68.8%) of studies that evaluated multiple model types. In total, 25 studies compared their NLP-based systems with current practice in their respective clinical settings and reported potential benefits, including improved data coverage and completeness, faster information extraction, and improved classification or prediction accuracy. No studies evaluated the utility or impact of their systems in real-world clinical practice. The most common challenges reported by authors were restricted access to clinical notes (n=39) and limited data (n=18).

**Conclusions:**

The application of NLP to clinical notes in oncology has expanded, but most studies focus on information extraction rather than downstream clinical tasks. Oncology NLP has the potential to advance cancer research and patient care, but barriers remain to robust evaluation and clinical deployment of promising tools. Emerging generative AI approaches will need to overcome these challenges to deliver real-world impact.

## Introduction

### Background

Cancer is a major cause of morbidity and mortality globally [1], with 19.3 million new cases and 10 million deaths reported in 2020 [1]. Incidence is projected to rise by 55% by 2040 due to population growth and aging [2]. Research leveraging real-world data is important to support prevention, early detection, and optimized treatment, and ultimately improve patient outcomes, including survival. Electronic health records (EHRs), digital profiles of patient histories created and managed by health care institutions, provide a valuable real-world data resource for cancer research and improve patient care.

While EHR systems have become increasingly available [3], only a small portion consists of structured data (eg, clinical codes, vital signs, clinical and laboratory measurements, and demographics) that can be easily extracted and analyzed using conventional statistical and machine learning methods. Most data (80%) exist in unstructured forms, including clinical notes, diagnostic reports (eg, pathology and radiology), and images [4], limiting usability [5]. Natural language processing (NLP)—a subfield of artificial intelligence (AI) that enables computers to understand, interpret, and generate human language—offers a promising approach to unlock insights from unstructured clinical narratives such as clinical notes and diagnostic reports, enabling their use in research and patient care.

While both diagnostic reports and clinical notes contain valuable information, they differ in complexity for NLP. Diagnostic reports are typically formal and standardized, making them relatively straightforward to process. In contrast, clinical notes are highly diverse due to variations in recording practices across clinicians and health care institutions [6]. They often feature incomplete sentences, poor punctuation, nonstandard abbreviations, shorthand, ambiguous terms, and spelling errors. These characteristics pose significant challenges for NLP processing, even with advanced methodological approaches such as pretrained language models (PLMs), for example, Bidirectional Encoder Representations from Transformers (BERT) [7-9], which dominated the general NLP domain since the introduction of the BERT model in 2018 [10].

However, recent advances in generative AI are reshaping the field of clinical NLP. Large language models (LLMs)—a subset of PLMs designed for generative tasks (eg, OpenAI’s GPT [11] and Meta’s LLaMA [12])—are transforming clinical NLP by enabling broader generalization with minimal task-specific fine-tuning. LLMs (GPT-4, Gemma3-27B, and DeepSeek-14B), applied using prompt engineering or task-specific fine-tuning, have demonstrated strong performance in extracting treatment histories [13], social and behavioral determinants of health (employment, housing, marital status, alcohol use, tobacco use, and drug use) [14], and neurofibromatosis type 1–relevant phenotypes [15] from clinical notes. Recent review studies highlight increasing interest in the use of LLMs with prompt-based strategies, including zero-shot and few-shot prompting, for information extraction (IE) [16,17], as well as for tasks such as information summarization, translation, and clinical communication [18].

Given their strong early performance, which has generated considerable interest within the NLP community, LLMs may emerge as a dominant approach, potentially replacing traditional supervised deep learning methods (eg, recurrent neural networks [RNNs], convolutional neural networks [CNNs], and BERT-based models). To better understand the value that LLMs add beyond established NLP approaches, we conducted a scoping review of NLP methods applied to cancer clinical notes prior to the widespread use of generative AI, providing a comprehensive overview of pre-LLM methods, their potential clinical utility, and the limitations and challenges likely to extend to generative AI.

Several reviews have examined the application of NLP to clinical notes before the adoption of LLMs; however, none have specifically focused on clinical notes as the primary text. Prior reviews have included clinical notes only as a subset of broader document categories. Only 35% (43/123), 22% (5/23), and 12% (2/17) of studies included in Wang et al [19], Li et al [20], and Gholipour et al [21], respectively, used clinical notes, often alongside other medical documents (eg, radiology and pathology reports). Sangariyavanich et al [22] included 17 studies but did not specify the proportion or extent of clinical note use. Furthermore, these reviews focused on one NLP task or the other, for example, IE [19,21], diagnostic classification [20], and prognostic classification [22]. Broader reviews by Wang et al [23], Sim et al [24], and Sheikhalishahi et al [25] covered studies, which included substantial volumes of clinical notes but were not cancer-specific, limiting their utility to the cancer domain. Additionally, these reviews only include studies published up to 2020, predating the widespread adoption of BERT-based PLMs. Notably, in Sheikhalishahi et al [25], only 3 of the 106 studies used deep learning approaches.

### Objectives

This review provides a comprehensive synthesis of NLP applications to clinical notes in cancer research prior to widespread experimentation with LLMs. Unlike prior review that included studies based solely on structured diagnostic reports, we restricted inclusion to studies involving clinical notes (exclusively or in combination with diagnostic reports or other documents), so our findings more closely reflect the distinctive challenges—including acquisition—and methodological choices associated with this particularly complex text. We also diverge from earlier reviews by imposing no restrictions on the NLP task, allowing a broader characterization of cancer-related use cases beyond conventional diagnostic or prognostic classification.

By systematically analyzing pregenerative AI methodologies, this review provides important benchmarks for assessing the real “value add” of LLMs, highlights the limitations of extractive, supervised approaches, and anticipates challenges that may need to be overcome. Specifically, our objectives are (1) to characterize the clinical notes used in NLP studies, including their sources and properties; (2) to identify NLP techniques (including annotation methods) used to analyze these notes and examine how these methodologies have evolved over time; (3) to determine the clinical applications of NLP in cancer research and patient care, including reported clinical impact; and (4) to highlight the challenges encountered by researchers in the field.

## Methods

This review follows the PRISMA-ScR (Preferred Reporting Items for Systematic Reviews and Meta-Analyses Extension for Scoping Reviews) [26].

### Working Definitions

We broadly defined NLP as the application of computational techniques to process and analyze unstructured clinical text. This encompasses a diverse range of methods, including domain-specific dictionaries, medical ontologies (eg, Unified Medical Language System [UMLS]), ontology-based tools (eg, MetaMap and Clinical Text Analysis and Knowledge Extraction System), handcrafted rules or search strings, rule-based tools (eg, ConText and NegEx), classical machine learning models (eg, support vector machine), neural networks (eg, RNN), PLMs (eg, BERT), and LLMs (a subset of PLMs distinguished by their larger parameter scale and enhanced capacity for broad generalization with minimal task-specific fine-tuning [eg, GPT]).

Clinical notes were defined as free-text narratives written by health care providers during patient encounters, documenting patient symptoms and signs, investigations, diagnoses, treatment, or treatment plans. They detail a patient’s social and medical history, disease progression, and outcomes. They are distinguished from diagnostic reports, in that they later provide results of diagnostic investigations or imaging studies, often objective and structured. Clinical notes may, however, contain descriptions and interpretations of diagnostic results from these reports.

### Search Strategy and Information Sources

We developed a three-concept search criterion covering (1) NLP, (2) EHR or electronic medical record, and (3) cancer or oncology. Predetermined key terms relating to these concepts were used to search MEDLINE through PubMed. These were further expanded by scanning the titles and abstracts of retrieved records. To avoid missing studies in which clinical notes were only one of several document types and therefore not mentioned in the title or abstract, we intentionally kept the EHR or electronic medical record concept broad. The final search criteria for all 4 databases are provided in Multimedia Appendix 1.

We searched MEDLINE (via PubMed), Embase, Web of Science, and Scopus for primary studies that applied NLP to process and analyze clinical notes to generate actionable information for cancer research or patient care. For PubMed, Embase, and Web of Science, we searched across all available fields. In Scopus, the search was limited to the title, abstract, and keywords fields. We used a mix of MeSH term mappings and exact phrase or term searching to balance the sensitivity and precision of the search. All searches were restricted to English-language publications from January 1, 2014, to March 8, 2024.

#### Inclusion Criteria

We included peer-reviewed journal papers and conference papers that (1) applied NLP to clinical notes—either exclusively or in combination with other medical documents (eg, pathology, radiology, colonoscopy, or other imaging reports); (2) focused on any part of the cancer care continuum, including screening, diagnosis, staging, treatment, surveillance, outcomes assessment, and risk factor identification or risk stratification; and (3) were conducted in any clinical setting (eg, primary care, outpatient clinics, emergency departments, and hospitals).

#### Exclusion Criteria

We excluded studies that used non-EHR documents (eg, patient-authored text in online health communities), studies using translated text (eg, from one language to English before applying NLP methods), reviews, editorials, commentaries, abstracts, letters, retracted papers, and veterinary studies.

### Study Selection

Study screening (title or abstract and full text) was completed in Covidence (Veritas Health Innovation Ltd), a web-based collaboration software platform that streamlines the production of systematic and other literature reviews. References identified through database searches were imported into Covidence, and duplicates were automatically removed.

Two authors (ABK and HRAE) independently assessed the papers for eligibility based on the title and abstract. Proportionate agreement (the proportion of times that reviewers agree on their assessments) was 96%. Class-specific agreement was 56.2% for the positive (include) class and 97.9% for the negative (exclude) class. Cohen κ, which measures the agreement between 2 reviewers (ABK and HRAE) adjusting for the possibility of agreement occurring by chance, was 0.54. Full-text papers were retrieved for studies that passed the title-abstract screening, and the same authors assessed the full texts for eligibility. Proportionate agreement was 81.5%. Class-specific agreement was 86.3% for the positive (include) class and 71.8% for the negative (exclude) class. Cohen κ was 0.58. At both stages, discrepancies were discussed and resolved through consensus, with reference to the predefined inclusion or exclusion criteria and the operational definitions of key concepts (NLP, clinical notes, and cancer or oncology). When consensus could not be reached, another author (GF or KL.) adjudicated.

### Data Extraction and Analysis

A data extraction template was created in Covidence and refined through several iterations until all authors agreed on the final version. Using this template, we extracted data across 37 predetermined variables, which can be classified into 5 categories: study metadata, clinical note characteristics, methods, applications, and challenges. Two authors (ABK and HRAE) extracted data from 10% of the papers. The extracted data were compared, and inconsistencies were discussed. Concordance was high and so the remaining papers were extracted by 1 reviewer (ABK). Extracted data were analyzed descriptively, providing counts and percentages.

### Study Quality Assessment

Given the scoping review methodology and our count-based analyses, a risk of bias or quality assessment was not performed [27].

## Results

### Search Results

**Figure 1.**
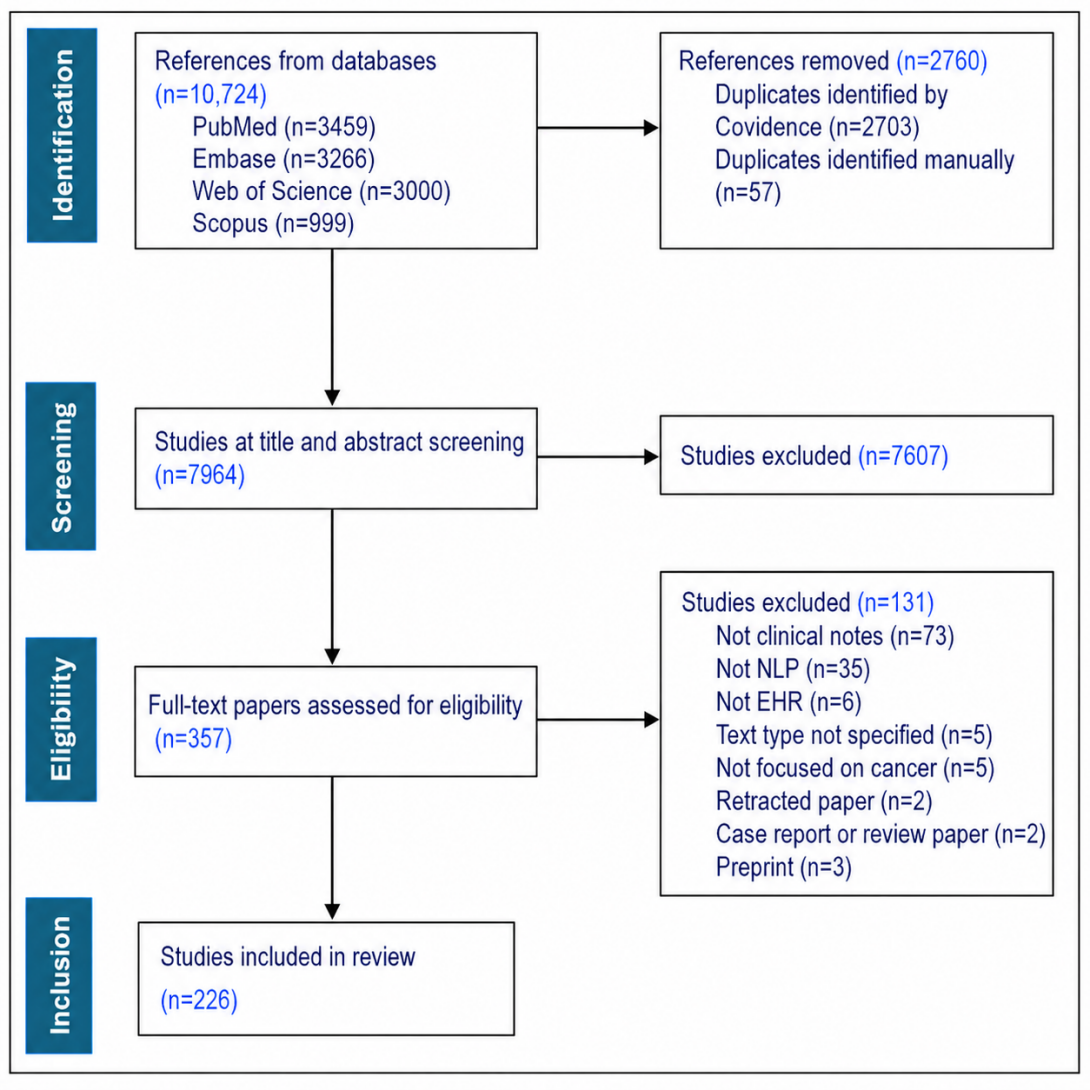
PRISMA diagram illustrating the study selection process and reasons for exclusion. EHR: electronic health record; NLP: natural language processing; PRISMA: Preferred Reporting Items for Systematic Reviews and Meta-Analyses.

Figure 1 shows the study selection process used to arrive at the included studies. A total of 10,724 records were identified from the databases. After removing duplicates, 7964 records were screened. Of these, 7607 were excluded at the title and abstract screening stage. In the full-text screening stage, 357 papers were assessed for eligibility, and 131 were excluded. Ultimately, 226 studies met the inclusion criteria.

### Distribution of Studies by Country

**Figure 2.**
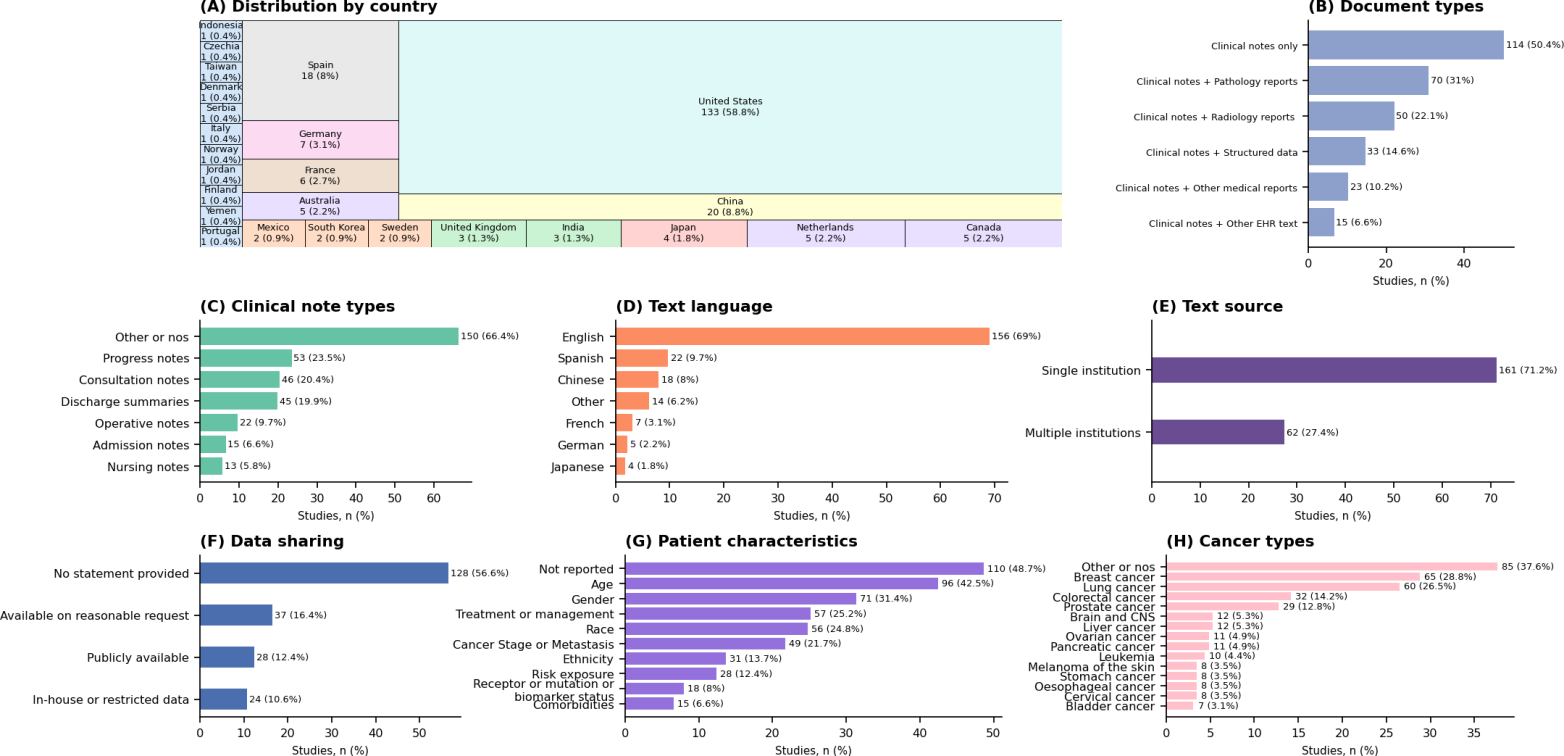
Characterization of included studies. (**A**) Distribution of studies by country of institution of affiliation of major authors. (**B**) Document type. (**C**) Clinical note type. (**D**) Language of clinical notes and other medical documents. (**E**) Heterogeneity of the sources of clinical notes and other medical documents. In total, 3 (1.3%) studies had insufficient information to determine the source of clinical notes. (**F**) Accessibility of data used by the studies (publicly available means that authors used a publicly available corpus [majority] or made their corpus publicly available). (**G**) Patient characteristics reported in studies. (**H**) Cancer types targeted by studies. CNS: central nervous system; nos: not otherwise specified.

Figure 2A illustrates the distribution of included studies based on the country of institution of affiliation of the major (first or corresponding) authors. The majority were from the United States (133/226, 58.8%), followed by China (20/226, 8.8%) and Spain (18/226, 8%).

### Characterization of Clinical Notes

Figure 2B shows the document types used across included studies. Out of 226 studies, 114 (50.4%) used clinical notes exclusively, while the remainder used clinical notes and other medical documents, primarily pathology and radiology reports. Progress notes (53/226, 23.5%), consultation notes (46/226, 20.4%), and discharge summaries (45/226, 19.9%) were the most common types of clinical notes used in included studies (Figure 2C). However, in 150 of the 226 (66.4%) studies, authors either used nonspecific terms to describe clinical notes (eg, oncology, urology, and cancer clinic notes) or did not specify the clinical note type. Most of the clinical notes were written in English (156/226, 69%), Spanish (22/226, 9.7%), and Chinese (18/226, 8%; Figure 2D).

Figure 2E illustrates heterogeneity in the sources of clinical notes and other medical documents used in the studies. Most studies (161/226, 71.2%) used documents from a single institution, while 27.4% (62/226) included multi-institution data from the same country. No study used documents from more than 1 country. Regarding data availability, 128 of 226 (56.6%) studies did not provide any statement on the accessibility of the corpora used. A few studies (37/226, 16.4%) indicated that their corpus could be made available upon reasonable request, and 12.4% (28/226) either used publicly available corpora (majority) or made their corpus publicly accessible (Figure 2F).

Nearly half of the studies (110/226, 48.7%) did not provide any information about the characteristics of the patients associated with the clinical notes they used. When reported, common characteristics included age (96/226, 42.5%), sex or gender (71/226, 31.4%), race (56/226, 24.8%), cancer therapy or management (57/226, 25.2%), and cancer stage or metastasis (49/226, 21.7%; Figure 2G). The most commonly studied cancers were breast (65/226, 28.8%), lung (60/226, 26.5%), colorectal (32/226, 14.2%), and prostate (29/226, 12.8%; Figure 2H).

### NLP Publications and Methods Used by Calendar Year

**Figure 3.**
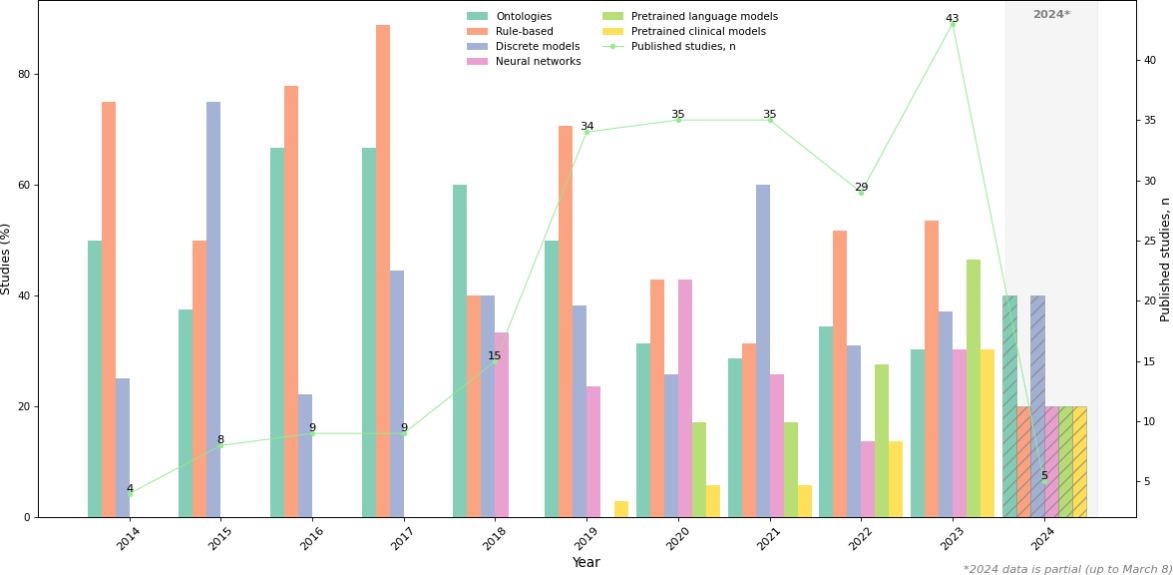
Model architectures used to analyze clinical notes over the years. Percentages are relative to the number of studies published in that year. The line graph depicts the number of published studies per year. *2024 is a partial year; it includes papers published from January 1, 2024, to March 8, 2024. It is common for researchers to use multiple methods from the same class or different classes (either as discrete models or in hybrid architectures), leading to double-counting. “Pretrained language models” refers to general-domain pretrained models (eg, BERT and GPT), while “pretrained clinical models” refers to models with domain-specific pretraining on clinical or biomedical text (eg, BioBERT, ClinicalBERT, and PubMedBERT). These categories are mutually exclusive. BERT: Bidirectional Encoder Representations from Transformers.

Figure 3 illustrates the number of studies published annually from January 2014 to March 2024, along with the NLP methods applied to clinical notes. The number of publications per year increased from 4 in 2014 to 43 in 2023.

NLP methods have evolved over time. Between 2014 and 2017, only ontologies, rule-based approaches, and discrete models were used. Studies using neural networks were first published in 2018, followed by PLMs in 2019 (Figure 3). While neural networks, including PLMs, have gained popularity since their introduction, ontologies, rule-based approaches, and discrete models remained the most prevalent approaches throughout the review period. However, rule-based approaches and ontologies were often used in hybrid workflows, serving specific preprocessing and postprocessing roles, rather than as standalone solutions. Out of 226 studies, only 7 (3.1%) and 27 (11.9%) exclusively used ontologies and rule-based methods, respectively.

### Fine-Grained Classification of NLP Methods

Ontologies were used in 87 of 226 (38.5%) studies, with domain-specific or customized dictionaries being the most common approach (42/87, 48.3%), followed by the UMLS at 41.4% (36/87; Table 1). These knowledge resources often supported machine learning and neural models by providing seed terms or domain expertise. Off-the-shelf tools such as MetaMap and Clinical Text Analysis and Knowledge Extraction System, which rely on UMLS mappings to analyze biomedical text, were also used.

**Table 1.** Breakdown of methods used in included studies (N=226)^a^.

Model architecture	Values (N=226), n (%))
Ontologies (n=87)	
Domain-specific dictionary	42 (48.3)
Unified Medical Language System	36 (41.4)
MetaMap	16 (18.4)
cTAKES^b^	10 (11.5)
NCBO^c^ BioPortal	7 (8)
MedTagger	3 (3.4)
Other	6 (6.9)
Rule-based (n=112)	
Rules or RegEx^d^	112 (100)
Discrete models (n=87)	
Support vector machine	29 (33.3)
Trees	28 (32.2)
Logistic regression	18 (20.7)
Conditional random fields	16 (18.4)
Clustering	15 (17.2)
Other	11 (12.6)
Naive Bayes classifier	5 (5.7)
K-nearest neighbors classifier	3 (3.4)
Linear regression	2 (2.3)
Neural networks (n=53)	
Recurrent neural network	34 (64.2)
Convolutional neural network	21 (39.6)
Feed forward neural networks	10 (18.9)
Capsule networks	1 (1.9)
Pretrained language models (n=41)	
BERT^e^	39 (95.1)
ChatGPT	1 (2.4)
Google Bard	1 (2.4)
Pretrained clinical models (n=23)	
Clinical BERT	23 (100)

a Number of model types per study: 91 (40.3%) studies used 1 model type, 92 (40.7%) studies used 2 model types, 26 (11.5%) studies used 3 model types, and 10 (4.4%) studies used 4 model types. Number of model subtypes per study: 76 (33.6%) studies used 1 model subtype, 72 (31.9%) studies used 2 model subtypes, 39 (17.3%) studies used 3 model subtypes, 25 (11.1%) studies used 4 model subtypes, and 4 (1.8%) studies used 5 model subtypes. Pretrained language models are general-domain pretrained models (eg, BERT and GPT), while pretrained clinical models are models pretrained on clinical or biomedical text (eg, BioBERT, ClinicalBERT, and PubMedBERT).

b cTAKES: Clinical Text Analysis and Knowledge Extraction System.

c NCBO: National Center for Biomedical Ontology.

d RegEx: a rule-based algorithm for negation detection in clinical text.

e BERT: Bidirectional Encoder Representations from Transformers.

Rule-based methods, including handcrafted rules and off-the-shelf tools such as clinical RegEx and ConText, were used in 112 of 226 (49.6%) studies (Table 1), making them the most prevalent, but rarely used in isolation. Rule-based approaches were used in 53 of 114 (46.5%) studies that analyzed clinical notes exclusively and in 64 of 112 (57.1%) studies that analyzed clinical notes in combination with other medical documents. Although the latter proportion was 10.6 percentage points higher, this difference was not statistically significant (2-proportion *z* test: *z*=−1.60; *P*=.11).

Discrete models, encompassing classical machine learning and statistical methods, were used in 87 of 226 (38.5%) studies (Table 1). The most common approaches under this category included support vector machines (29/87, 33.3%), tree-based models including random forest (28/87, 32.1%), logistic regression (18/87, 20.7%), conditional random fields (16/87, 18.4%), and clustering algorithms (15/87, 17.2%). Conditional random field was often applied as a classification layer in neural models like long short-term memory and CNN.

Neural networks featured in 53 of 226 (23.5%) studies, with RNN (34/53, 64.2% ) and CNN (21/53, 39.6%), being the most popular in this category (Table 1). RNNs were dominated by long short-term memory architectures.

PLMs were used in 41 of 226 (18.1%) studies. These were primarily BERT-based models, with only 2 of the 41 (0.9%) studies [28,29] using LLMs (ChatGPT and Google Bard; Table 1). Pretrained clinical models—BERT-based models pretrained on clinical or biomedical corpora (eg, Bio_ClinicalBERT)—were used in 23 of 226 (10.2%) studies (Table 1). Among 23 studies that implemented pretrained clinical models, 16compared clinical domain pretrained models to general domain pretrained models. Clinical domain models outperformed general domain models in 11 of 16 (68.8%) studies, while general domain models performed better in the remaining 5 studies (Multimedia Appendix 2).

### Methods for Non-English Corpora

Out of 226 studies, 70 (40%) developed models for non-English clinical notes. Of these, 59 (84.3%) implemented language-specific pipelines built from rules and classical machine learning with engineered features, including some hybrid combinations. Pretrained approaches were present but less common and not mutually exclusive across studies: language-specific pretrained models in 11 of 70 (15.7%) studies, multilingual pretrained models in 7 of 70 (10%) studies, language-specific biomedical or clinical pretrained models in 6 of 70 (8.6%) studies, and language-adapted models in 3 of 70 (4.3%) studies. Language-adapted models typically consisted of models pretrained in English and then further trained on the target language (Multimedia Appendix 3).

In total, 12 studies compared multiple model families. Language-specific biomedical or clinical pretrained models most often yielded the best performance (n=4) [30-33], followed by language-specific pretrained models (n=3) [34-36] and language-adapted pretrained models (n=2) [37,38]. In the remaining 3 studies, the best-performing models were a biomedical or clinical pretrained model [39], a language-specific model [40], and a multilingual pretrained model [41].

### Text Representation Methods

**Figure 4.**
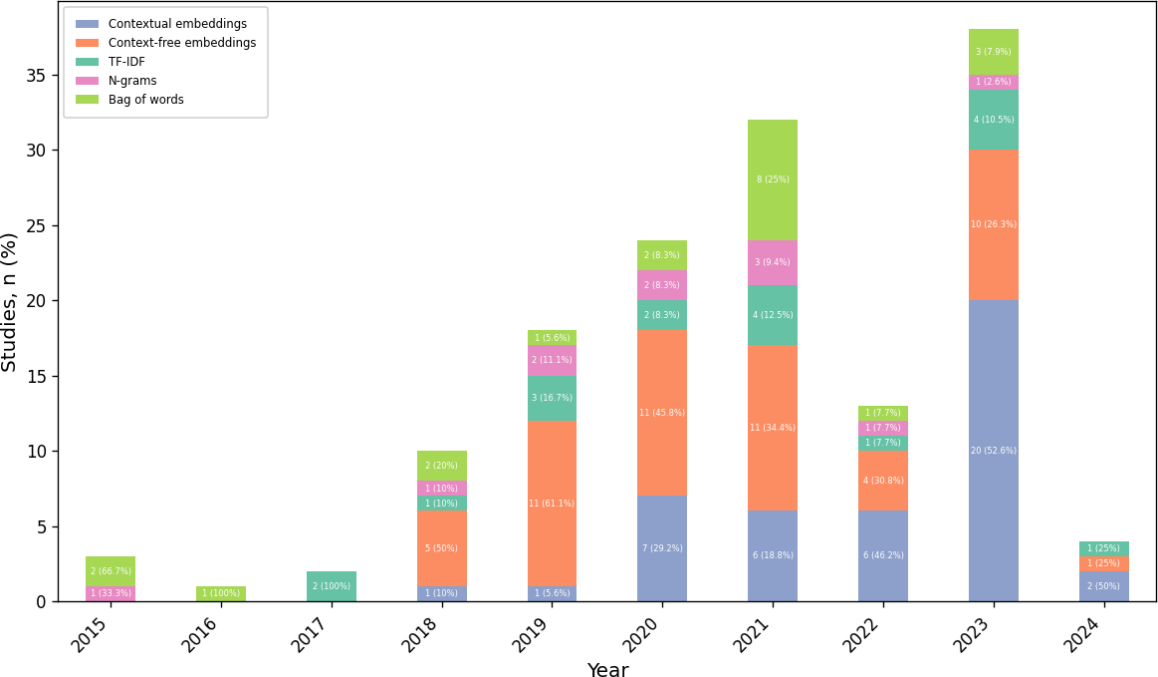
Text representation and embedding methods (n=120). Context-free embeddings include Word2Vec, FastText, and GloVe. N-grams include continuous bag of words, skip-gram, bigrams, and trigrams. Groups are not mutually exclusive—studies may appear in more than 1 category. TF-IDF: term frequency-inverse document frequency.

Figure 4 illustrates the text representation and vectorization methods used in the studies. Out of 226 studies, 120 (53.1%) used at least 1 representation method. From 2015 to 2017, statistical methods including bag of words, n-grams, and term frequency-inverse document frequency were prevalent. In 2018, context-free embeddings (one fixed vector for each word or token regardless of the context in which it is used, eg, Word2Vec, GloVe, and FastText) and contextual embeddings (a new vector assigned to each word or token depending on the surrounding context, eg, BERT and GPT) were introduced and became the predominant approaches. It was common for studies to test multiple embedding methods to identify the best-performing approaches.

### Size of Labeled Data Used to Train and Evaluate NLP Systems

**Figure 5.**
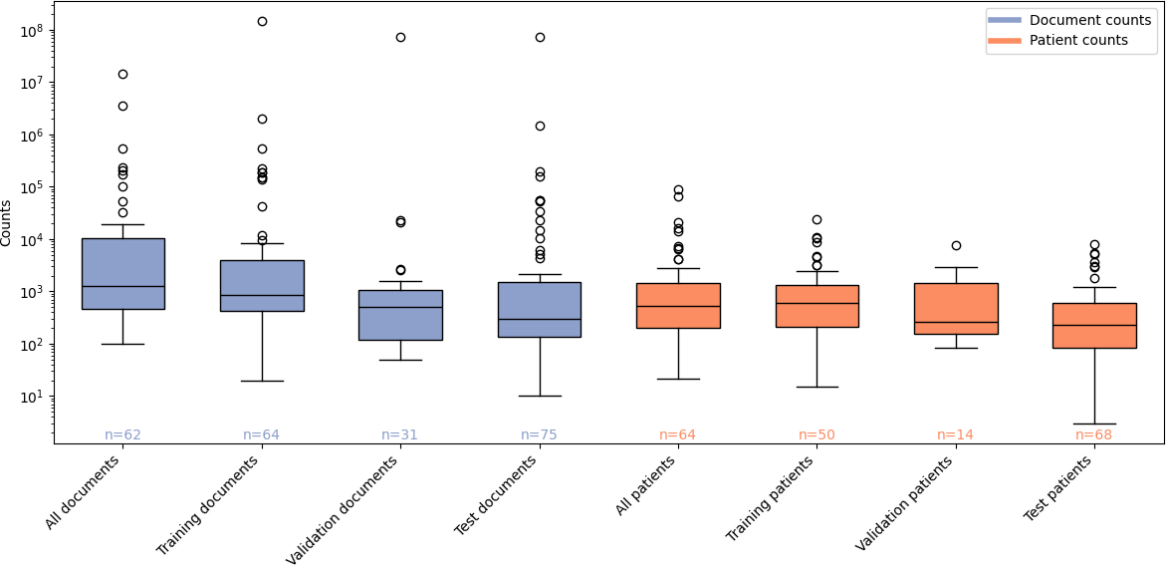
Size of the data used in model development and evaluation. Documents refer to entire clinical notes or reports or sentences (a small number of studies reported corpus size in sentences). To cater for instances where train or test split was not specified, we report total data sums (ie, all documents and all patients) as provided by the authors. The number below each boxplot indicates the count of studies reporting data size in that category.

Figure 5 shows the data size (clinical notes, with or without additional medical documents, and patients) used to train and evaluate NLP systems. The median number of documents per partition was fewer than 1000, and the median number of patients associated with these notes was also under 1000. For example, the median number of training documents, test documents, training patients, and test patients was 838 (IQR 439-3905), 300 (IQR 120-1504), 606 (IQR 202-1337), and 231.5 (IQR 86-599), respectively.

Training and test sets were generally created through random splits, except in 3 studies where the test cohort came from a slightly different patient population (prospective palliative radiation cohort vs metastatic cancer retrospective registry–based cohort) [42], a different but overlapping time period with the training cohort [43], a different nonoverlapping time period with the training cohort [44], or where the test cohort had a shorter follow-up time than the training cohort (4 vs 5 years) [45].

### Annotation Methods for Reference Corpus

The majority of studies (181/226, 80.1%) trained and evaluated their systems on corpora that were manually annotated by humans. Few studies (7/226, 3.1%) trained models using weakly supervised labels but evaluated them on human-curated labels. A considerable proportion of studies (38/226, 16.8%) either relied on existing labels within the EHR (eg, *International Classification of Diseases* or ICD codes) or developed unsupervised systems, for which manual annotation was not applicable. A summary of annotation methods is provided in Multimedia Appendix 4.

### Implementation Type and Evaluation

Table 2 summarizes model implementation type, evaluation metrics, and whether models were externally evaluated. Most studies (179/226, 79.2%) developed new models or retrained or fine-tuned an existing one, while 19.5% (44/226) used existing models without retraining. The latter group included studies that used off-the-shelf tools such as MetaMap or repurposed existing models for new extraction tasks.

Evaluation metrics varied by task, with the most commonly reported being recall (155/226, 68.6%), precision (153/226, 67.7%), *F*_1_-score (136/226, 60.2%), accuracy (44/226, 19.5%), area under the receiver operating characteristic curve (40/226, 17.7%), and specificity (30/226, 13.3%). While metrics such as recall, precision, and *F*_1_-score were widely used and therefore suitable for summarization, variability in clinical corpora and tasks precluded comparison on NLP methods. Only 21 of 226 (9.3%) studies evaluated their systems on external corpora.

**Table 2.** Model implementation and evaluation (N=226)^a^.

Implementation or evaluation	Values, n (%)
Implementation type	
New model	179 (79.2)
Existing model	44 (19.5)
Reported evaluation metrics	
Recall	155 (68.6)
Precision	153 (67.7)
*F*_1_-score	136 (60.2)
Accuracy	44 (19.5)
AUC-ROC^b^	40 (17.7)
Specificity	30 (13.3)
Cohen κ	5 (2.2)
Cosine similarity	3 (1.3)
Mean average precision	2 (0.9)
Other	44 (19.5)
External evaluation	
Yes	21 (9.3)
No	158 (69.9)

a Some studies lacked sufficient information to assess external evaluation; for example, those that used existing tools had their detailed data documented elsewhere.

b AUC-ROC: area under the receiver operating characteristic curve.

### Clinical Applications of NLP

**Figure 6.**
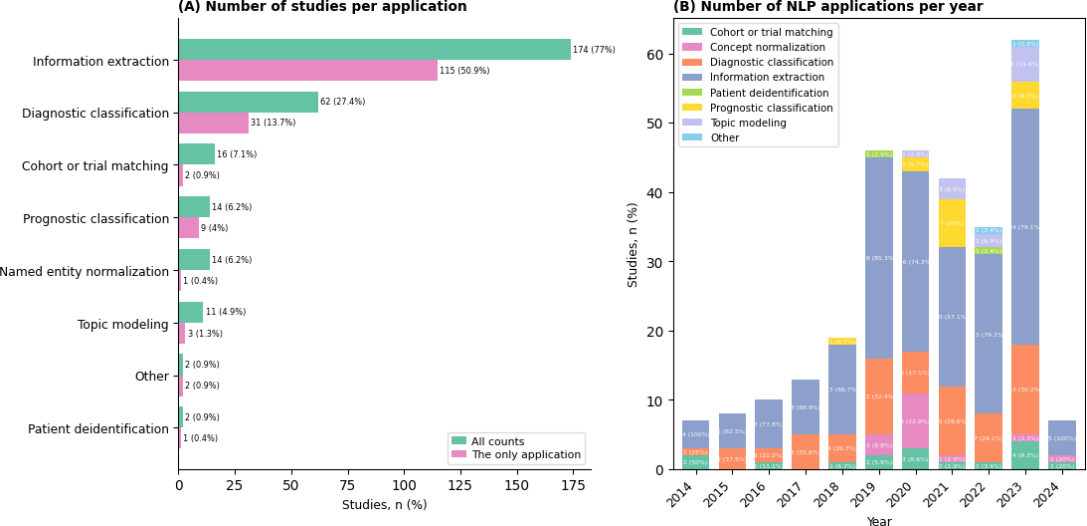
NLP clinical applications with clinical notes. (**A**) Number of studies per clinical application. (**B**) Number of clinical applications per year (percentages are relative to the number of papers published in that year). Diagnostic classification refers to document-level or patient-level classification tasks, for example, distinguishing between notes with metastasis and those without metastasis. Prognostic classification refers to predicting that some clinical event of interest will occur within a specified time period in the future, for example, lung cancer recurrence 2 years following lobectomy. NLP: natural language processing.

Figure 6A summarizes the clinical applications of NLP to clinical notes. IE was the most common task, with 77% (174/226) of the studies. In 50.9% (115/226) of the studies, NLP was exclusively used for IE. Diagnostic classification was performed in 62 of 226 (27.4%) studies, while trials or cohort matching was the goal in 16 of 226 (7.1%) studies. Other notable applications included prognostic classification (n=14), concept normalization (n=14), and topic modeling (n=11). It was not uncommon, however, for a study to undertake multiple tasks, often with the output of one task feeding into subsequent tasks.

A subset of studies (n=15) that focused on IE also extracted temporal information. Some studies formulated this task as a document-time relation (DocTimeRel) classification, where events were assigned a temporal relation to the document creation time (before, after, overlap, or before or overlap) [46-48]. Others used an event-date relation classification formulation, classifying event-time pairs as before, after, overlap, or before or overlap [49] or directly linking events to their corresponding dates through contextual pairing [50,51]. One study constructed patient-level temporal timelines by assigning events to coarse temporal bins (way before admission, before admission, admission, after admission, and discharge) and then temporally ordered them within and across documents [52]. Less complex approaches included proximity- or context-based methods (linking events to nearby date mentions using dependency parsing and rule-based contextual heuristics) [53-58] or simply classifying identified events into broad temporal categories such as current, history, future, or unknown [59,60].

Figure 6B shows the evolution of clinical NLP applications over time. IE remained the predominant task throughout the years, followed by diagnostic classification. Newer applications introduced after 2018 include concept normalization, prognostic classification, and topic modeling. Task chaining, where the output of one task is used as the input for downstream tasks, was common in studies that went beyond IE. For example, in 2014, there were 4 publications of NLP applied to clinical notes. All 4 (100%) studies used NLP to extract information of some kind, 2 (50%) studies used the extracted information to match patients to clinical trials, 1 (25%) study used the extracted information for diagnostic classification, and 1 (25%) study had IE as the end point. Discrete models were almost exclusively used for these downstream tasks.

### System Deployment Stage and Clinical Impact

Of the 226 reviewed studies, 224 (99.1%) developed proof-of-concept systems that were evaluated only in research settings rather than deployed in routine clinical practice. One study piloted their system in clinical practice [61], while another described the use of an NLP-based system that had just been integrated in clinical use [62].

Since most studies were evaluated only as research implementations (ie, no real-world deployments), clinical impact was not evaluated. However, 25 of the 226 (11.1%) studies compared their systems with current practice in their research implementation. These studies reported benefits such as improved data coverage (identified more patients with the relevant attribute than from structured data alone) and completeness (curated further variables not available as structured data) [63-75], taking less time to extract relevant information [29,61,76-82], fewer clinician man-hours for certain tasks (eg, fewer clinicians needed to complete clinical audits) [29], and higher classification or prediction accuracy compared with human experts or existing methods [76,83,84]. One study that described an IE system in routine use [62] focused on characterizing use patterns, including which clinical specialties used the system and for what purposes.

### Challenges and Limitations Reported by the Authors

Table 3 details challenges and limitations faced by researchers applying different NLP techniques to clinical notes. Common challenges were single-institution corpora (39/226, 17.3%), limited data (18/226, 8%), incomplete EHR data (14/226, 6.2%), label imbalance (12/226, 5.3%), rules or dictionary not comprehensive or generalizable (9/226, 4%), and word sense and abbreviation disambiguation (6/226, 2.7%). Overall, authors reported a range of challenges, some unique to the task, corpora, or methodological approach.

**Table 3.** Challenges and limitations reported in studies^a^.

Challenge or limitation	Values, n (%)
Single institution corpus [37,42,44,61,70,72,73,75,77,79,82,84-111]	39 (17.3)
Limited data [32,50,57,61,62,65,79,90,92,104,111-117]	18 (8)
Incomplete recording in the EHR^b^ [42,57,74,78,81,94,98,103,109,118-122]	14 (6.2)
Label imbalance [31,38,44,73,82,98,102,104,123-126]	12 (5.3)
Negation detection and resolution [41,74,97,119,126-131]	10 (4.4)
Dictionary or rules not comprehensive or generalizable [65,66,92,119,120,132-135]	9 (4)
Word sense or abbreviation disambiguation [48,130,131,136-138]	6 (2.7)
Variability in terminology used to describe the same concept [78,120,136,139]	4 (1.8)
Spelling errors or typos [90,130,137,140]	4 (1.8)
Imbalanced data [57,102,105,141]	4 (1.8)
Use of speculative language [117,128,136]	3 (1.3)
Use of nonstandard terminology [90,128,142]	3 (1.3)
Rarity of concepts of interest [41,45,143]	3 (1.3)
Institutional differences in documentation style or note structure [42,81,117]	3 (1.3)
Quality of human annotations [51,80]	2 (0.9)
Multilingualism in text [79,128]	2 (0.9)
Temporal reasoning (current vs historical events) [129,138]	2 (0.9)
Short notes or sentences (insufficient context for context-dependent models) [72,144]	2 (0.9)
Model computationally expensive [38]	1 (0.4)
Distant (intersentence) relations [124]	1 (0.4)
Frequency of co-occurrence of unrelated concepts [143]	1 (0.4)
Long execute-response time [145]	1 (0.4)
Very long documents ( >512 token limit for BERT^c^-based models) [125]	1 (0.4)
Significant n-gram method insensitive to evolution of patient’s notes over time and between patients [146]	1 (0.4)
Resolution of patient and nonpatient references [97]	1 (0.4)
Nonstandard date formats [57]	1 (0.4)

a Negation detection and resolution includes detecting the negation itself, distant negations, and resolving the scope of the negation. Limited data encompass the following: small corpus, only a small number of patients associated with those notes, and small annotated or labeled notes for model development and evaluation. Imbalanced data refer to instances where notes are overrepresented by text from one patient group (eg, private insurance vs noninsured). Label imbalance is when one label of interest (eg, a certain biomarker) is more prevalent in the notes, hence, easily learned by the model at the expense of other labels (biomarkers). Quality of human annotations is where human annotated corpora for model training and evaluation are erroneous.

b EHR: electronic health record.

c BERT: Bidirectional Encoder Representations from Transformers.

## Discussion

### Summary of Main Findings

Research applying NLP to clinical notes in the cancer domain grew substantially during the review period, rising from 4 publications in 2014 to 43 in 2023, likely driven by the increasing availability of digital records and advances in scalable NLP methods. However, most studies relied on English language (156/226, 69%) and single-institution (161/226, 71.2) datasets. The majority of studies originated from the United States (133/226, 58.8%), which aligns with trends in clinical NLP publishing in which the United States dominates [147]. Almost half of the studies (110/226, 48.7%) provided no information on the characteristics of patients whose clinical notes were used, while 56.6% (128/226) did not provide a statement on data sharing, limiting interpretability and reproducibility. The most commonly studied cancers (breast, lung, colorectal, and prostate) likely reflect their prevalence in the United States and hence dedicated EHR systems, which in turn increases the availability of clinical notes.

NLP methods for processing clinical notes evolved from exclusively ontology-based, rule-based, and discrete models (2014‐2017) to hybrid approaches incorporating neural networks and PLMs such as BERT (2018‐2024). Only a few studies applied LLMs, with publications starting from October 2023. Contextual embeddings have become increasingly prevalent, reflecting the wider adoption of pretrained models. Most studies used small single-institution datasets (<1000 documents or <1000 patients), likely due to challenges in accessing clinical notes. Annotation methods were mostly manual. A subanalysis of non-English corpora studies showed that the majority (59/70, 84.3%) implemented language-specific, nonpretrained models. Domain-specific pretrained clinical models were superior to other model types in the majority (11/16, 68.8%) of studies across both English and non-English corpora. Only 9.3% (21/226) of studies evaluated their systems on external datasets.

Most studies (174/226, 77%) focused on IE. A subset of these used the extracted information in downstream tasks, but the majority (115/226, 50.9%) focused solely on IE. In total, 15 studies extracted temporal information from clinical notes using various approaches, including DocTimeRel classification, event-time relation classification, and proximity- or context-based methods. No studies evaluated clinical impact following implementation, but several studies compared their systems to current practice in their respective settings (eg, manual review of notes in clinical audits) and demonstrated potential clinical utility. The most common challenge in clinical NLP was restricted access to sufficient clinical notes, reported by 17.3% (39/226) of studies.

### Evolution of NLP Methods for Clinical Notes

NLP methods for clinical notes have become more diverse over time. While new deep learning–based techniques have gained popularity, they have largely complemented rather than replaced traditional methods such as rules and ontologies, resulting in widespread adoption of hybrid architectures. Prior reviews that included substantial volumes of clinical notes reported similar findings, namely, the predominance of rule-based methods alongside increasing use of hybrid architectures that combine rules with machine learning or neural networks [24,25]. However, a review of NLP applied to diagnostic (radiology) reports reported slightly different findings, with rule-based and classical machine learning methods being prevalent but often used as baselines against which deep learning approaches were compared [148].

The continued use of rule-based approaches for clinical notes likely reflects the unique challenges posed by these documents, which often require substantial preprocessing before neural models can be applied, as well as postprocessing to structure model outputs into clinically meaningful formats. The overall prevalence of rule-based methods may also partly reflect the inclusion of semistructured diagnostic reports, which—owing to their templated design and restricted, domain-specific vocabulary—are generally more amenable to rule-based processing [149]. Combining knowledge resources with deep neural models, on the other hand, may reflect authors’ efforts to enhance the explainability of predictions made by these complex networks, given the importance of explainability in health care AI [150,151] and evidence from prior work that integrating knowledge into deep learning may improve explainability [152].

Text representation methods have evolved alongside machine learning models. Earlier NLP approaches commonly relied on discrete word representations, such as term frequency-inverse document frequency and n-grams [153]. Our review shows that context-free word embeddings (eg, Word2Vec, GloVe, and FastText) were the most widely used, typically with classical machine learning models. The results also suggest that these approaches are increasingly being complemented or replaced by contextual embeddings derived from transformer-based models, which represent words as vectors that capture richer semantic and syntactic relationships.

### Trends in NLP Clinical Applications

NLP applications to clinical notes focused predominantly on IE, accounting for over three-quarters of included studies, with comparatively limited use in downstream clinical decision-making tasks. This emphasis reflects both the pragmatic advantages and the perceived safety of IE. By structuring free-text data into clinically meaningful variables, IE enables expert oversight, produces interpretable intermediate outputs, and supports a broad range of secondary applications, including diagnostic or prognostic modeling, cohort identification, and decision support [154]. In contrast, approaches that predict outcomes directly from unstructured text without an explicit IE step are often less transparent, constrain the incorporation of domain knowledge, and are typically optimized for a single task [155].

### Potential Clinical Impact of NLP

Although none of the included studies evaluated the direct clinical impact of NLP systems on patient care following research implementation, several studies compared their systems with current clinical practice as part of their evaluation. These comparisons demonstrated the potential of NLP to support tasks such as IE, clinical auditing, and diagnostic or prognostic classification. However, most studies (161/226, 71.2%) relied on small, single-institution datasets, raising concerns about generalizability, as such models often perform less well when applied to more representative or external datasets due to differences in both population characteristics and data structure. Without extensive evaluation across diverse datasets, there remains limited evidence of real-world effectiveness, thereby impeding adoption into routine clinical use.

Beyond technical performance, the application of NLP systems to high-risk tasks, such as cancer diagnosis or risk prediction, is subject to stringent regulatory oversight as medical devices [156,157]. These regulatory requirements, together with challenges in integrating NLP systems into existing clinical workflows [158], further hinder translation into routine clinical care and help explain the limited real-world impact observed across studies.

### Challenges and Opportunities in Advancing Clinical NLP

Our findings indicate that restricted access to clinical data remains the dominant barrier in oncology NLP. Access to clinical corpora is complicated by multiple barriers, including national data protection regulations governing privacy and confidentiality (eg, the General Data Protection Regulation [159] in the European Union and the Health Insurance Portability and Accountability Act [160] in the United States), additional institutional governance restrictions imposed to mitigate disclosure risk and legal liability [161], and technical obstacles such as EHR interoperability [161]. This is compounded by limited data sharing practices, with many studies providing no clear data availability statement or listing data as “available on reasonable request,” a practice that often creates substantial practical barriers, including low response rates and protracted negotiations that effectively limit access. As a result, researchers have to rely on small, single-institution datasets, resulting in proof-of-concept systems with limited generalizability.

Limited data accessibility undermines reproducibility, hinders meaningful comparison across studies, prevents the establishment of standardized benchmarks for performance evaluation, and reinforces reliance on small, single-institution datasets. Collectively, these challenges derail real-world deployment of clinical NLP systems.

Several methodological approaches have attempted to mitigate data scarcity, each with notable limitations. Transfer learning through clinical PLMs (eg, ClinicalBERT) is constrained by training on relatively small and institutionally narrow corpora, reflecting the same access limitations they aim to overcome, which can result in suboptimal performance on downstream tasks [162,163]. Publicly available deidentified datasets curated for clinical NLP shared tasks (eg, Cancer Text Mining Shared Task [164]) face similar limitations, being small and single-center.

More recently, LLMs have shown promise in mitigating data scarcity by enabling zero-shot or few-shot learning, thereby reducing dependence on large, manually annotated corpora [165,166]. However, LLMs introduce additional challenges, including the propagation of embedded biases [167], privacy breaches [168], model obsolescence and drift [168], hallucination and confidently stated falsehoods [169,170], and substantial computational and environmental costs. These shortcomings can be detrimental to clinical practice, for example, by systematically underrecommending investigations, procedures, or treatments for underrepresented patient groups. Therefore, research on LLMs should also focus on addressing these ethical concerns in addition to technical performance and generalizability.

Model-centric privacy-preserving approaches, such as federated learning, where models are trained locally and aggregated without sharing raw data [171], offer a potential pathway toward multi-institutional collaboration without direct data transfer. However, practical deployment remains challenging, requiring compatible infrastructure, sustained institutional partnerships, and strategies to manage data heterogeneity and site imbalance, which can bias global models toward dominant contributors and degrade performance for underrepresented populations [172]. Related techniques, such as differential privacy, may further reduce reidentification risk but introduce trade-offs between privacy protection and model utility that must be carefully managed [173].

Beyond algorithmic solutions, structural and policy-level interventions are likely to be critical. National initiatives, such as those implemented in Denmark, where clinical notes are rigorously deidentified and made accessible within secure research environments [69], demonstrate the feasibility of balancing privacy protection with research utility. Broader adoption of such frameworks, alongside clearer institutional agreements that permit sharing of rigorously deidentified clinical text and accompanying code, could substantially improve reproducibility and accelerate progress in oncology NLP. Furthermore, to support open science in oncology, NLP future studies should adopt more transparent reporting of data access conditions, and where feasible, publicly release the code, alongside clear governance mechanisms to balance reproducibility with patient privacy.

### Limitations of the Review

This review has several limitations. First, approximately half of the included studies analyzed clinical notes alongside more structured medical documents such as pathology or radiology reports. These document types differ substantially in linguistic complexity, with diagnostic reports often being more templated and semistructured compared to free-text clinical notes such as progress notes or discharge summaries. As a result, the NLP methods and challenges reported in such studies may not be fully representative of those encountered when analyzing highly unstructured clinical narratives.

Second, we were unable to determine the proportion of clinical notes versus other document types in each study, as this was rarely reported. While we distinguished document types where possible, inconsistent reporting limited further quantification of these documents. Consequently, our findings reflect the broader landscape of clinical text processing in oncology rather than exclusively characterizing NLP applied to highly unstructured clinical notes. Nonetheless, we provide a more faithful representation of pregenerative AI methodological choices and challenges associated with clinical notes, as all included studies incorporated clinical notes. In addition, we could not systematically compare model performance across studies due to substantial heterogeneity in corpora and NLP tasks.

Third, the predominance of studies authored by researchers from the United States (133/226, 58.8%), primarily using local datasets, may have introduced some geographical and system-level bias. Our findings are therefore more reflective of the US health care context, including workflows, documentation styles, clinical note structures, and data access provisions.

Finally, Cohen κ for title or abstract screening (0.54) and full-text screening (0.58) indicated moderate interrater agreement. This primarily reflects challenges in operationalizing eligibility criteria. In particular, disagreement frequently arose from ambiguity in how studies described their textual data sources, as some authors used the term “clinical notes” broadly to refer to any textual medical document, including diagnostic reports. This was exacerbated by limited methodological detail in the abstract, making it difficult to determine whether clinical notes were included. Despite this, class-specific agreement for exclusions at title or abstract screening was high (97.9%), while agreement for included studies improved substantially at full-text screening (86.3%) once detailed information was available. The moderate κ values could therefore be partly attributed to class imbalance inherent to evidence synthesis, as most records are excluded at the title or abstract, and κ adjusts for agreement expected by chance.

### Conclusions

This review establishes a comprehensive pregenerative AI baseline for NLP applied to clinical notes in oncology. Over the past decade, research volume increased substantially, and methods evolved from rule-based approaches to hybrid architectures incorporating rules and neural networks, including PLMs. However, most studies focused on IE rather than diagnosis or prognostication, relied on small single-institution datasets, and lacked external validation. While several systems demonstrated superior performance compared to current practice in research settings, significant barriers to clinical deployment remain, including limited generalizability, poor reproducibility, and restricted data access. Emerging generative AI approaches will need to address these barriers, as well as broader ethical challenges, to enable the translation of NLP systems into clinical settings for real-world impact.

## Supplementary material

10.2196/73481 Multimedia Appendix 1 Search criteria.

10.2196/73481 Multimedia Appendix 2 Studies included in the review and variables extracted.

10.2196/73481 Multimedia Appendix 3 Models for non-English corpora.

10.2196/73481 Multimedia Appendix 4 Annotation methods for reference corpus. Annotation granularity ranged from the entity or concept level to the patient level, including sentence, document section, and document levels. No information: no description of annotation methods (studies that used existing tools, detailed methods described elsewhere).

10.2196/73481 Checklist 1 PRISMA-ScR checklist.

## Abbreviations

AI: artificial intelligence
BERT: Bidirectional Encoder Representations from Transformers
CNN: convolutional neural network
EHR: electronic health record
ICD: International Classification of Diseases
IE: information extraction
LLM: large language model
NLP: natural language processing
PLM: pretrained language model
PRISMA-ScR: Preferred Reporting Items for Systematic Reviews and Meta-Analyses Extension for Scoping Reviews
RNN: recurrent neural network
UMLS: Unified Medical Language System
