# Supplementary material for: Natural Language Processing of Clinical Notes for Cancer Research and Patient Care Prior to Widespread Adoption of Generative AI: Scoping Review
Source: JMIR AI. 2026 May 14;5:e73481. doi: 10.2196/73481 (PMC13175237; doi:10.2196/73481)
Supplement: Multimedia Appendix 1 [file ai-v5-e73481-s001.docx]

**PubMed**

**Search**: (((((((((Natural Language Processing) OR (NLP)) OR ("Language model*")) OR (("Named entity") AND ("recognition" OR "extract*"))) OR ("Coreference resolution")) OR (("Concept*" OR "Relation" OR "Information") AND ("extract*"))) OR (("Text" OR "Information") AND ("summar*" OR "simplif*" OR "retriev*" OR "mining" OR "translat*" OR "classif*"))) AND (((((((Electronic Health Record) OR (Electronic Medical Record)) OR ("EHR")) OR ("EMR")) OR ("Medical reports")) OR (("medical" OR "clinical" OR "nurs*" OR "admission" OR "discharge") AND ("notes" OR "narratives" OR "summaries" OR "text"))) OR (("Patholog*" OR "Radiolog*" OR "Imaging" OR "Oncolog*") AND ("reports")))) AND (((((((Cancer) OR (Neoplasm)) OR (Neoplas*)) OR (Malignan*)) OR (Tumour)) OR (Tumor)) OR (Carcinoma))) NOT (((((("Systematic review")) OR ("Scoping review")) OR ("Overview")) OR ("Review")) OR ("Update")) Filters: English, Exclude preprints, from 2014/1/1 - 2024/3/8

**Embase**

**Query**: (('natural'/exp/mj OR natural) AND ('language'/exp/mj OR language) AND ('processing'/exp/mj OR processing) OR nlp OR 'language model*' OR ('named entity' AND ('recognition'/mj OR 'recognition' OR 'extract*')) OR 'coreference resolution' OR (('concept*' OR 'relation' OR 'information'/mj OR 'information') AND 'extract*') OR (('text' OR 'information'/mj OR 'information') AND ('summar*' OR 'simplif*' OR 'retriev*' OR 'mining'/mj OR 'mining' OR 'translat*' OR 'classif*'))) AND (electronic AND ('health'/mj OR health) AND record OR (electronic AND medical AND record) OR 'ehr' OR 'emr' OR 'medical reports' OR (('medical' OR 'clinical' OR 'nurs*' OR 'admission' OR 'discharge') AND ('notes' OR 'narratives' OR 'summaries' OR 'text')) OR (('patholog*' OR 'radiolog*' OR 'imaging' OR 'oncolog*') AND 'reports')) AND ('cancer'/mj OR cancer OR 'neoplasm'/de OR neoplas* OR malignan* OR 'tumour'/de OR 'tumor'/de OR 'carcinoma'/de) AND ('article'/it OR 'conference abstract'/it OR 'conference paper'/it) AND [english]/lim AND [abstracts]/lim AND [2014-2024]/py AND [embase]/lim

**Web of Science**

# Searches

1: ((((((ALL=(Natural Language Processing)) OR ALL=(NLP)) OR ALL=(“Language model*”)) OR ALL=((“Named entity”) AND (“recognition” OR “extract*”))) OR ALL=(“Coreference resolution”)) OR ALL=((“Concept*” OR “Relation” OR “Information”) AND (“extract*”))) OR ALL=((“Text” OR “Information”) AND (“summar*” OR “simplif*” OR “retriev*” OR “mining” OR “translat*” OR “classif*”))

2: ((((((ALL=(Electronic Health Record)) OR ALL=(Electronic Medical Record)) OR ALL=(“EHR” )) OR ALL=(“EMR”)) OR ALL=(“Medical reports”)) OR ALL=((“medical” OR “clinical” OR “nurs*” OR “admission” OR “discharge”) AND (“notes” OR “narratives” OR “summaries” OR “text”))) OR ALL=((“Patholog*” OR “Radiolog*” OR “Imaging” OR “Oncolog*”) AND (“reports”))

3: ((((((ALL=(Cancer)) OR ALL=(Neoplasm)) OR ALL=(Neoplas*)) OR ALL=(Malignan*)) OR ALL=(Tumour)) OR ALL=(Tumor)) OR ALL=(Carcinoma)

4: (((((#1) NOT ALL=(“Systematic review”)) NOT ALL=(“Scoping review”)) NOT ALL=(“Overview”)) NOT ALL=(“Review”)) NOT ALL=(“Update”)

5: (((((#2) NOT ALL=(“Systematic review”)) NOT ALL=(“Scoping review”)) NOT ALL=(“Overview”)) NOT ALL=(“Review”)) NOT ALL=(“Update”)

6: (((((#3) NOT ALL=(“Systematic review”)) NOT ALL=(“Scoping review”)) NOT ALL=(“Overview”)) NOT ALL=(“Review”)) NOT ALL=(“Update”)

7: #4 AND #5 AND #6

**Scopus**

( ( TITLE-ABS-KEY ( {Natural Language Processing} ) ) OR ( TITLE-ABS-KEY ( {NLP} ) ) OR ( TITLE-ABS-KEY ( {Language model*} ) ) OR ( TITLE-ABS-KEY ( ( {Named entity} ) AND ( {recognition} OR {extract*} ) ) ) OR ( TITLE-ABS-KEY ( {Coreference resolution} ) ) OR ( TITLE-ABS-KEY ( ( {Concept*} OR {Relation} OR {Information} ) AND ( {extract*} ) ) ) OR ( TITLE-ABS-KEY ( ( {Text} OR {Information} ) AND ( {summar*} OR {simplif*} OR {retriev*} OR {mining} OR {translat*} OR {classif*} ) ) ) AND PUBYEAR > 2013 AND PUBYEAR < 2025) AND ( ( TITLE-ABS-KEY ( {Electronic Health Records} ) ) OR ( TITLE-ABS-KEY ( {Electronic Medical Records} ) ) OR ( TITLE-ABS-KEY ( {EHR} ) ) OR ( TITLE-ABS-KEY ( {EMR} ) ) OR ( TITLE-ABS-KEY ( {Medical reports} ) ) OR ( TITLE-ABS-KEY ( ( {medical} OR {clinical} OR nurs* OR {admission} OR {discharge} ) AND ( {notes} OR {narratives} OR {summaries} OR {text} ) ) ) OR ( TITLE-ABS-KEY ( ( Patholog* OR Radiolog* OR Imag* OR Oncolog* ) AND ( {reports} ) ) ) AND PUBYEAR > 2013 AND PUBYEAR < 2025 ) AND ( ( TITLE-ABS-KEY ( {Cancer} ) ) OR ( TITLE-ABS-KEY ( {Neoplasm} ) ) OR ( TITLE-ABS-KEY ( Neoplas* ) ) OR ( TITLE-ABS-KEY ( Malignan* ) ) OR ( TITLE-ABS-KEY ( {Tumour} ) ) OR ( TITLE-ABS-KEY ( {Tumor} ) ) OR ( TITLE-ABS-KEY ( {Carcinoma} ) ) AND PUBYEAR > 2013 AND PUBYEAR < 2025 ) AND NOT ( ( TITLE-ABS-KEY ( {Systematic review} ) ) OR ( TITLE-ABS-KEY ( {Scoping review} ) ) OR ( TITLE-ABS-KEY ( {Overview} ) ) OR ( TITLE-ABS-KEY ( {Review} ) ) OR ( TITLE-ABS-KEY ( {Update} ) ) AND PUBYEAR > 2013 AND PUBYEAR < 2025 ) AND ( LIMIT-TO ( DOCTYPE , "ar" ) OR LIMIT-TO ( DOCTYPE , "cp" ) AND ( LIMIT-TO ( LANGUAGE , "English" ) )
