# Supplementary figures and images for: Natural Language Processing of Clinical Notes for Cancer Research and Patient Care Prior to Widespread Adoption of Generative AI: Scoping Review
Source: JMIR AI. 2026 May 14;5:e73481. doi: 10.2196/73481 (PMC13175237; doi:10.2196/73481)

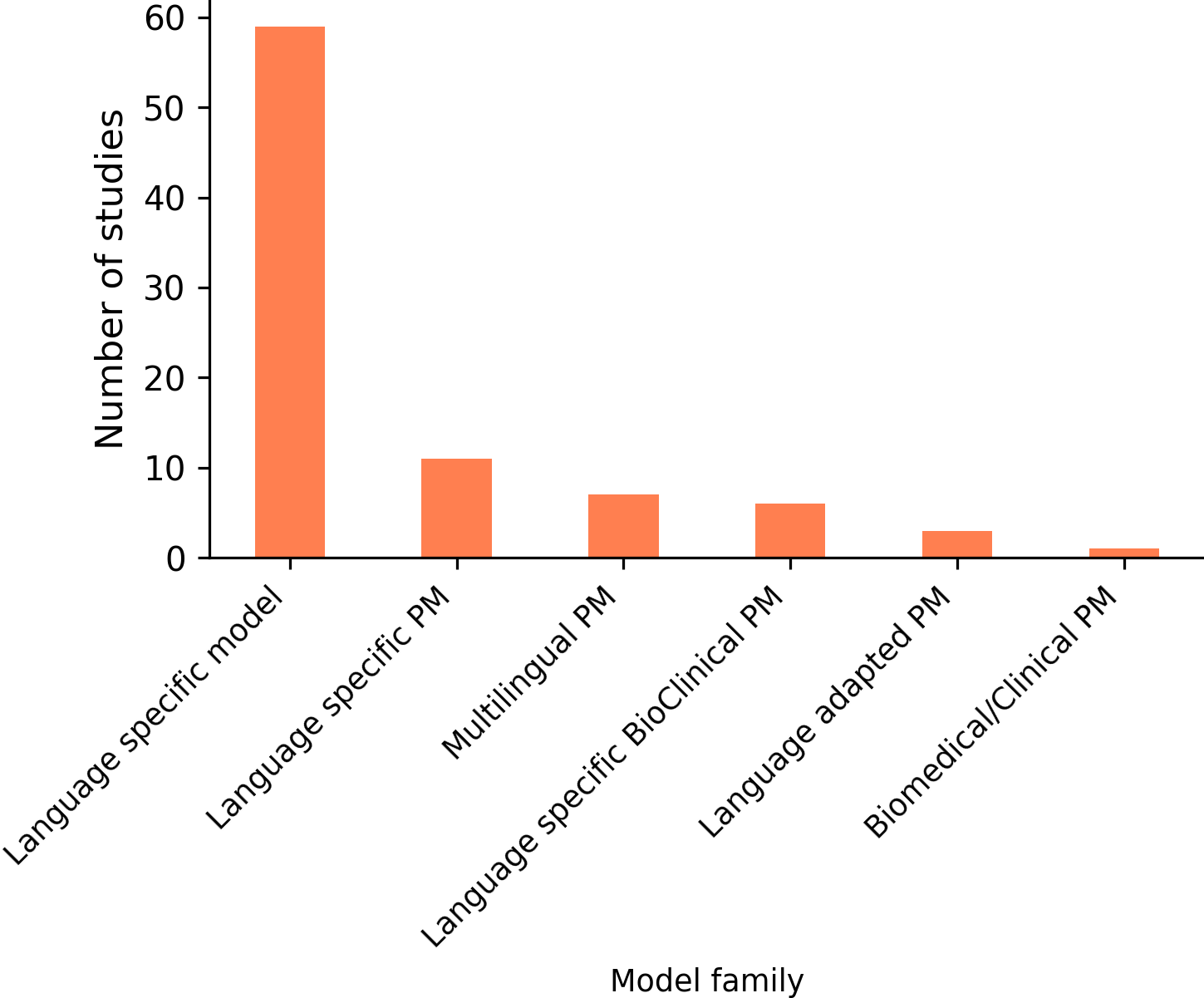

Supplement: Multimedia Appendix 3 [file ai-v5-e73481-s003.png]

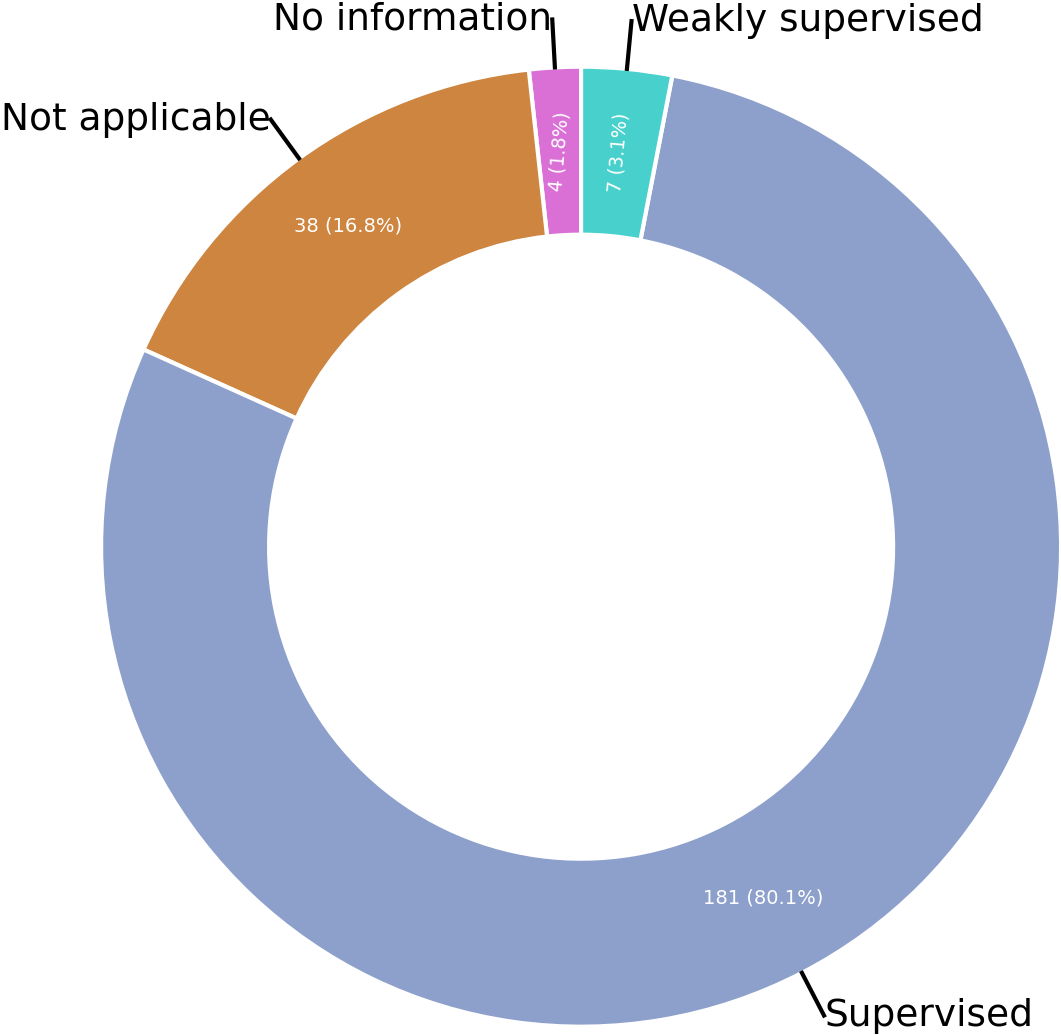

Supplement: Multimedia Appendix 4 [file ai-v5-e73481-s004.png]
